# Increased biological and cathodic hydrogen production using a novel integrated thermophilic fermenter and dual anion exchange membrane bioelectrochemical system

**DOI:** 10.1016/j.mex.2022.101770

**Published:** 2022-06-23

**Authors:** Nadali Alavi, Monireh Majlessi, Nazak Amanidaz, Mirzaman Zamanzadeh, Mohammad Rafiee

**Affiliations:** a Environmental and Occupational Hazards Control Research Center, Shahid Beheshti University of Medical Sciences, Tehran, Iran; b Department of Environmental Health Engineering, School of Public Health and Safety, Shahid Beheshti University of Medical Sciences, Tehran, Iran; c Department of Environmental Health Engineering, School of Public Health, Tehran University of Medical Sciences, Tehran, Iran

**Keywords:** Biohydrogen, Cathodic H_2_, Inhibitors recovery, Bipolar electrodialysis, Bio electrochemical, Double anion exchange membrane

## Abstract

Many researchers are interested in utilizing renewable and sustainable energy made by exoelectrogenic bacteria during electrodialysis for the separation of minerals and organic matters from aqueous environments. The aim of this study was to develop a novel thermophilic fermenter and dual anion exchange membrane bioelectrochemical system for separating biohydrogen production inhibitors from the thermophilic fermenter and thereby increasing biological and cathodic hydrogen production by food waste and wastewater.

• Using this innovative system the biohydrogen production inhibitors were separated and nutrients (for example ammonium), alkalinity, buffering capacity and pH were preserved in the bioreactor at the same time, led to higher biological and cathodic hydrogen production.

## Graphical abstract

**Figure:**
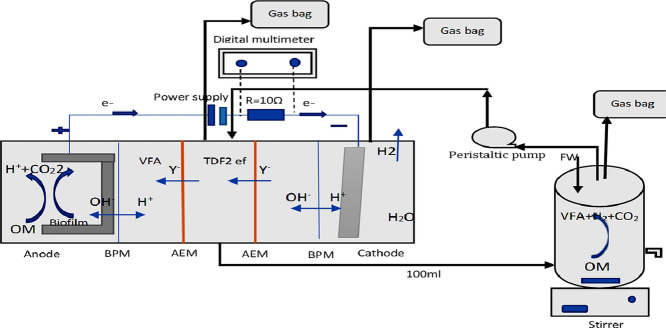

Specifications table

**Table:** 

Subject Area;	Energy
More specific subject area;	Biologial and cathodic hydrogen production
Method name;	Integrated thermophilic fermenter and dual anion exchange membrane bioelectrochemical system
Name and reference of original method;	Alavi, N., Majlessi, M., Amanidaz, N., Zamanzadeh, M., Rafiee, M., Gholizadeh, A., Mirzaee, S.A., Mokhtari, M., 2021. Enhanced biological hydrogen production through the separation of volatile fatty acids and ammonia based on microbial bipolar electrodialysis during thermophilic dark fermentation. J. Clean. Prod. 129887.
Resource availability	https://www.sciencedirect.com/science/article/abs/pii/S0959652621040579

## Experimental design

### Experimental thermophilic fermenter and dual anion exchange membrane bioelectrochemical system setup

**Fig. 1:**
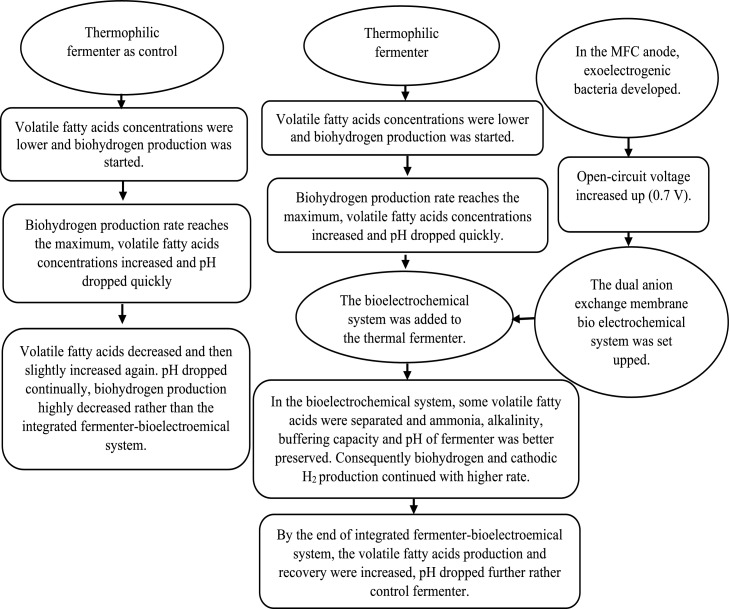
The investigation procedures.

This system consists of two parts; thermophilic fermentation and dual anion exchange membrane bioelectrochemical system, and each of them was set up separately before combination (Fig. 1).

### Thermophilic fermenter setup

Fermentation operations were carried out in a 0.5 L cylindrical airtight Plexiglas reactor [1]. The rectors were double-walled, and hot water was circulated between reactors walls to keep the temperature of the fermenters at thermophilic condition (55 °C). The magnetic stirrers were used to uniform fermenters liquid mixture. The total volume and working volume of fermenters were 0.5 and 0.45 L. Tedlar bags (SKC.3L) were connected to the fermenters for collecting biohydrogen gas.

### The microbial fuel cell system setup and biofilm growth on the anode

**Fig. 2:**
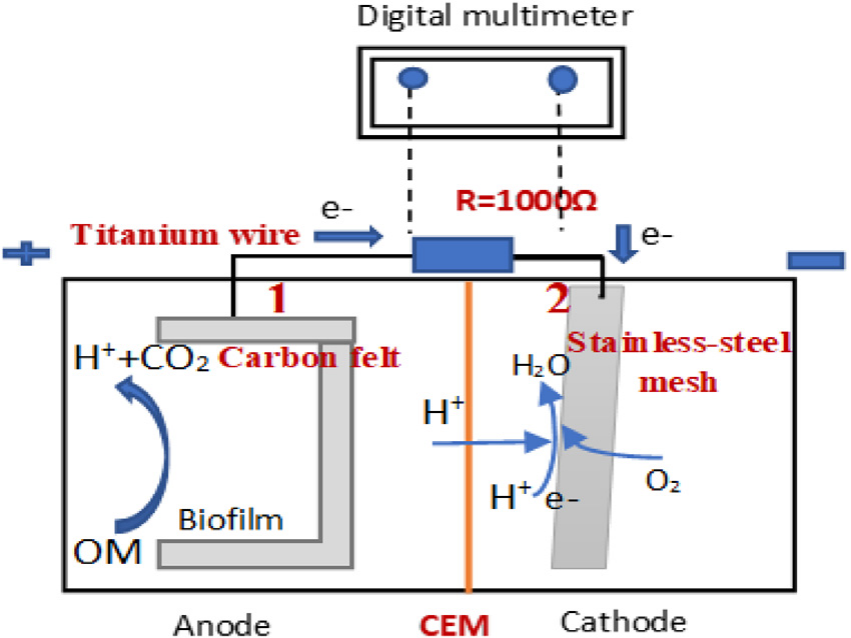
The microbial fuel cell system.

Before setting up the dual anion exchange membrane bioelectrochemical system, we set up and operated the system as a microbial fuel cell containing anode and cathode chambers as shown in Figs. 2 and S3.

**Fig. 3:**
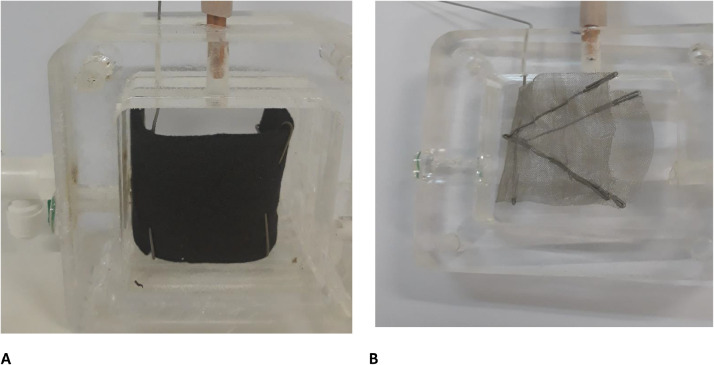
The U-shaped carbon felt (A), and V-shaped stainless-steel mesh (B) as anode and cathode electrodes.

**Fig. 4:**
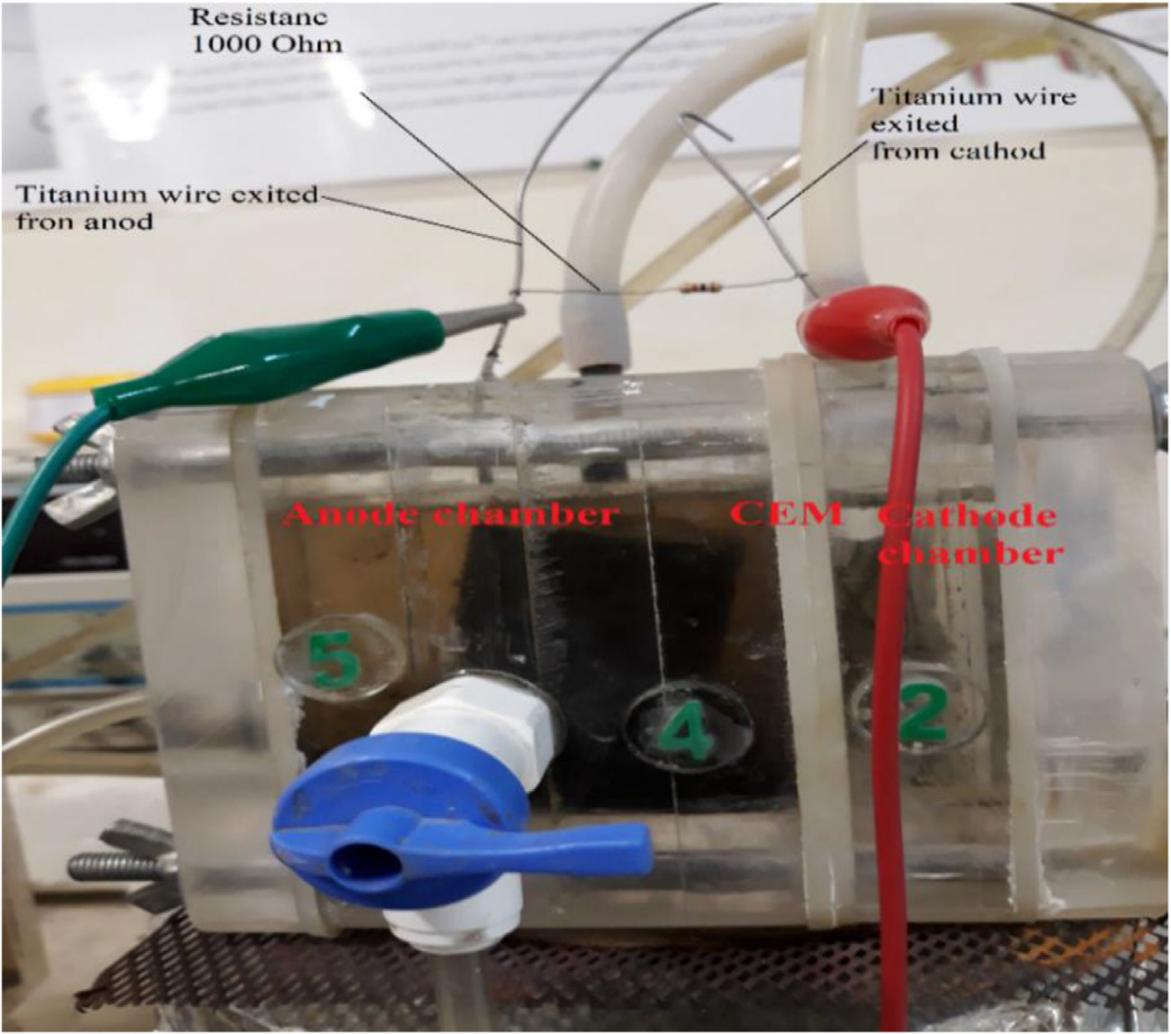
The image of the MFC system.

Anode electrode was carbon felt with 3.5 cm × 10 cm 0.5 cm dimensions and cathode electrode was a stainless-steel mesh, 4 cm × 10 cm. A U-shaped anode electrode and a V-shaped cathode electrode were used as shown in Fig. 3. The titanium wires were used among them to shape electrodes, collect electrons from the anode and conduce them toward the cathode. Rubber pipes were connected to the anode in order to nitrogen gas sparging as shown in Fig. 4. Electrochemically active bacteria were grown on carbon felt as anode electrode (Fig. S6). After two months and reaching a stable electricity generation by anode, 700 mV open-circuit voltage, the anode electrode was used in the dual anion exchange membrane bioelectrochemical system.

**Fig. 5:**
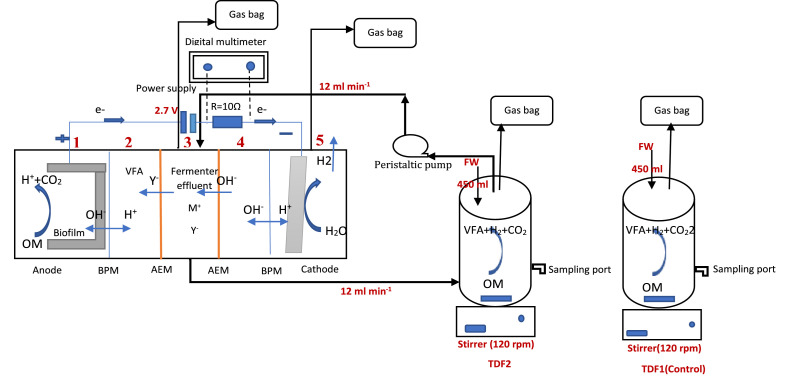
Schematic of the integrated thermophilic fermenter and dual anion exchange membrane bioelectrochemical system. Y^−^, ions; M‏, cations; chamber 1 is anode chamber; chamber 2 is volatile fatty acids separation chamber; chamber 3 is dilution chamber, chamber 5 is cathode chamber.

Subsequently, other membranes were added. The location of the membranes, the external resistance and the direct current power supply in the dual anion exchange membrane bioelectrochemical system are shown in Fig. 5. More details have been presented in our previous work [2].

### The dual anion exchange membrane bioelectrochemical system setup

**Fig. 6:**
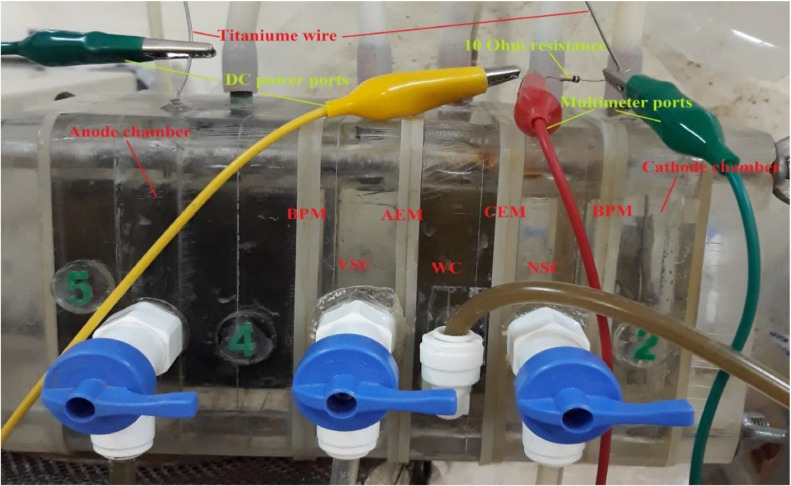
The image of the dual anion exchange membrane bioelectrochemical system.

As depicted in Fig. 6, this system was an airtight rector made of Plexiglass. Two bipolar membranes (BPM) were placed at the inner sides of the anode and cathode chamber at a distance of 2 mm from the electrodes. Two anion exchange membranes (AEM) were placed between the BPMs at a distance of 2.5 cm from each other and from two BPMs. The membranes and electrodes were shown in **Fig. S1**. A volatile fatty acids separation chamber was placed between BPM and AEM, near the anode chamber. The middle chamber between two AEMs was a diluted chamber that a fermenter liquid mixture was circulated in it. The inner dimension of the anode chamber was 6.5 × 6.5 × 6.5 cm^3^ (200 mL useful volume), and for others were 6.5 × 6.5 × 2.5 cm^3^ (80 mL useful volume). The sparging of nitrogen gases, electrolytes feeding and solution sampling were conducted through rubber tubes on each chamber. The substrate concentration of 70,000 mg COD L^−1^ was used in the thermal fermenters. The external voltage (2.7 V) was used to overcome system resistance.

### Feedstock and inoculum

Anaerobic sludge from the wastewater treatment plant digester of Tehran was used as inoculum. After sieving with a 2 mm diameter mesh to remove particles, in order to kill methanogens, the sludge was pre-heated at 100°C for 4 h and 15 min for using as the fermenters [3] and anode inoculum respectively [4]. Finally, the sludges were cooled to room temperature. Domestic wastewater from clarifier effluent after being amended with sodium acetate was used in order to feed the anode biofilm. The food waste was set according to Rajaeifar et al study [5], including vegetable residues, 54%; fruit peel, 30%; meat, 2%, boiled rice, 10% and bread; 5%. Then the food waste was grounded and sieved by mesh 0.5 mm to remove particles (Fig. S2) and was stored at -20 °C till use. The pre-heated sludge and raw food waste characteristics were according to Table 1.

**Table 1:** The pre-heated sludge and raw food waste characteristics.

Chemical properties	Pre-heated sludge	Food waste
Total Solids (TS)	40,700 mg L^−1^	179,000 g L^−1^
Volatile Solids (VS)	24,600 mg L^−^	167,800 g L^−1^
Volatile Solids / Total Solids ratio (VS/TS)	0.6	0.94
chemical oxygen demand (COD)	41,600 mg L^−1^	199,500 mg L^−1^
Total Kjeldahl Nitrogen (TKN)	3000 mg L^−1^	30 mg L^−1^
ammonium (NH_4_^+^)	950 mg L^−1^	2500 mg L^−1^
pH	8.9	4.3

### Thermophilic fermenters operation

According to Fig. 1, two thermophilic fermenters were operated, one of them was considered as control. The 400 mL of diluted food waste and 50 mL pre-heated sludge were used in thermophilic fermenters. Nutrient stock solution [6] was made as Table 2 and 12 mL of it was added to the thermophilic fermenters.

**Table 2:** Nutrient stock solution characteristic.

Materials	Concentrations (g L^−1^)
KH_2_PO_4_	100
NH_4_HCO_3_	200
MgSO_4_.7H_2_O	10
MnSO_4_.7H_2_O	1.5
Na_2_MoO_4_-2H_2_O	1
NaCl	1
CaC_l2_.2H_2_O	1
FeCl_2_	0.278

In order to remove oxygen from the fermenters solution after feeding, N_2_ was sparged into the fermenters for 15 min. The biohydrogen production rates, volatile fatty acids, ammonia concentrations, total solids, volatile solids, chemical oxygen demand and alkalinity concentrations were determined in both fermenters at times of 6, 8, 12 and 24 h. In the control run (70 C run), after the biohydrogen production rate reached a peak value, subsequently dropped quickly due to the accumulation of volatile fatty acids.

### The dual anion exchange membrane bioelectrochemical system operation

After biohydrogen production began to decrease as a result of volatile fatty acids accumulation in the fermenters, the bioelectrochemical system was integrated with one thermal fermenter in order to remove volatile fatty acids from fermenter mixed liquid. Another fermenter was considered as the control as shown in Fig. 4. The anodic electrolyte of the bioelectrochemical system was refilled every 48 h to provide sufficient food for exoelectrogenic bacteria. However, the external voltage was used to overcome the system internal resistance of BPM as well as produce cathodic H_2_ through reducing H^+^ ions in the cathode chamber [7]. A direct current (DC) power supply RXN-202D was used to supply 2.7 V in addition to the amount produced by the anode. The voltage between electrodes was recorded each 5 min using a digital multimeter (109 N, APPA). Two multimeter probs were connected on either side of the 10 Ω external resistance as shown in Fig. 5, 6. The 0.2 M NaCl was used as an electrolyte (pH 6.5–6.6) in the chambers of 2, 4, 5 (Fig. 5). Tedlar bags were used to collect hydrogen gas from fermenters, diluted chamber and cathode chamber. All bioelectrochemical system chambers and fermenters were purged with N_2_ before each run in order to create an anaerobic condition. The fermenter mixed solution was entered into the diluted chamber as shown in Fig. 5. Mesophilic temperature (35 °C) was utilized at the anode chamber and the thermophilic temperature was utilized at the fermenters (55 °C).

### Chemical and electrochemical analysis

Chemical, physical and electrochemical analysis and methods were according to Table 3.

**Table 3:** Chemical, physical and electrochemical analysis.

Variation	Experimental and analysis method
Volatile fatty acids concentrations	Gas chromatography (GC) with FID detector (Agilent 6890) as explained by Zhang and Angelidaki [7].
Hydrogen concentrations	GC with TCD detector (Agilent 7890) [7].
Hydrogen volumes	After gathering biogas in Tedlar bags, the volume of biogas was measured by liquid displacement method (**Fig. S7**) and reported under 0 °C, 1 atm [8]
Alkalinity	Titration method [9].
TKN	Semi-micro-kjeldahl method [9].
NH_4_^+^	Titration method [9].
COD	Closed reflux colorimetric method [9].
Total solid (TS)	Total solids dried at 103–105°^C^[9].
Volatile solid (VS)	volatile solids ignited at 550°C methods [9]
pH	WTW PHm 9310

## Method validation

### Volatile fatty acids separation and biohydrogen and cathodic hydrogen production

Followed by the bioelectrodialysis system was connected to the fermenter, the concentration of volatile fatty acids was highly decreased at first (until 10 h), later hydrogen-producing bacteria (HPB) was further stimulated to produce volatile fatty acids because some acids were removed from the fermenter solution where the HPBs exist. This led to increasing biohydrogen production rather than the control run as mentioned in Table 2. Volatile fatty acids production were increased up to 3.2 and 4.5 times rather than control run until 24 and 36 h (Table 2). Volatile fatty acids concentration was raised up to 0.51 g L^−1^ in the volatile fatty acids separation chamber at 24 h. Tang et al. [10] and Arslan et al. [11] increased acetate production 1.3 and 1.4 times using conventional electrodialysis integrated to the fermenter by utilizing high external energy [12].

**Fig. 7:**
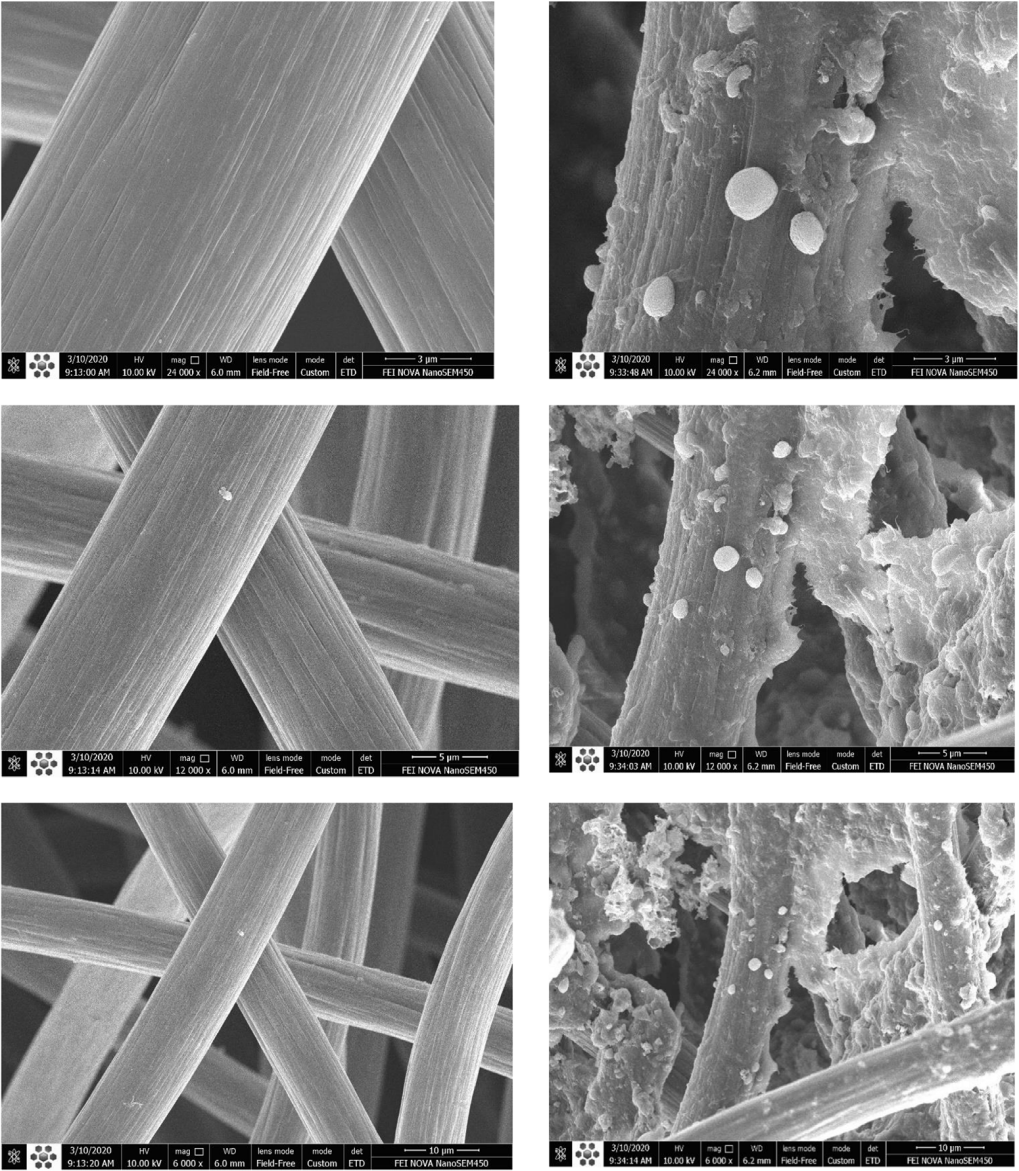

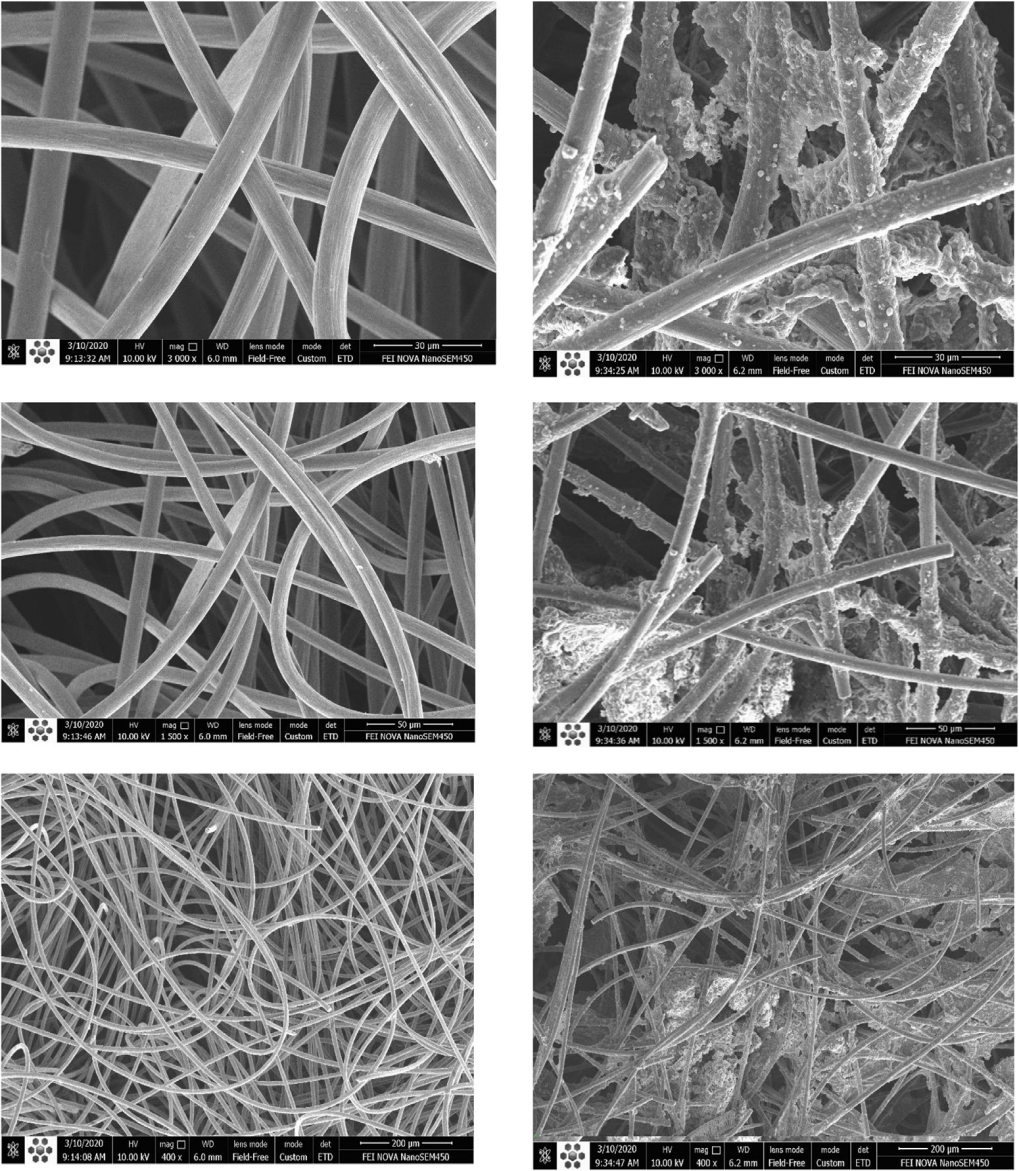
Different magnifications of carbon felt as anode electrode before and after biofilm growth on it.

Biohydrogen production was 632 mL L^−1^ higher rather than the control run, according to Table 4. This was because of volatile fatty acids separation, preserve nutrient (ammonium), higher alkalinity and pH by prevention of the passage of ammonium and other cations through the AEM from the dilute chamber towards cathode side. Furthermore, the ammonium and acetic acid concentrations were increased and this may be led to producing a higher concentration of ammonium acetate, which is often used with acetic acid to produce a buffer solution [13]. In addition, OH^−^ ions that were generated by BPM, may be entered towards dilute chamber. The higher concentrations of cations in diluted chamber maybe increase the OH^−^ ions pass towered diluted chamber. These lead to higher alleviate pH reduction in this system and thereby increased biohydrogen production. In our work, biohydrogen production yield was enhanced to 63.15 mL mg^−1^ versus 50.5 mL mg^−1^ in control. Hassan et al. [14] increased biohydrogen yields to 33.68 mL mg^−1^ using conventional electrodialysis while higher external voltage was used rather than our work. Furthermore, in our system, 50 mL cathodic hydrogen (**Fig. S5**) was produced after 36 h. Zhang and Angelidaki [7] obtained 18 mL of cathodic hydrogen for 48 h. Fig. 7 is show the carbon felt as anode electrode before and after biofilm growth on it.

**Table 4:** Volatile fatty acid concentration and cumulative biohydrogen production during various configurations (dashed line is related to MBED connection time).

Runs	Time (h)	Ac (mg/L)(±SD)	Pr (mg/L)(±SD)	Bu (mg/L)(±SD)	T VFAs (mg/L)(±SD)	Cumulative H_2_ production (mL/L)
70C	0	192.6 (±9)	24 (±3)	32 (±3)	248.6 (±15)	2879 (± 32)
	6	263.7 (±20)	16.3 (±3)	67.8 (±4)	347.8 (±27)	
	8	186 (±8)	11 (±2)	263.6 (±15)	460.6 (±25)	
	10	213.9 (±19)	35.2 (±3)	476 (±26)	725.1 (±60)	
	12	180.1 (±7)	33.4 (±4)	332.9 (±23)	546.4 (±34)	
	24	144.3 (±6)	63.2 (±5)	507 (±43)	714.5 (±54)	
70-2AEM	0	193 (±8)	40.2 (±6)	41.9 (±65)	274.2 (±20)	3511 (±35)
	6	263.6 (±16)	56.3 (±8)	61.5 (±8)	381.45 (±32)	
	8	334.7 (±43)	72.4 (±10)	81.1 (±15)	488.2 (±68)	
	10	258.7 (±46)	39.7 (±11)	19.5 (±3)	317.9 (±60)	
	12	866.9 (±94)	49.5 (±8)	64.3 (±6)	980.7 (±108)	
	24	1816 (±134)	57.6 (±10)	440.4 (±63)	2314 (±207)	
	36	2511.7 (±112)	59.7 (±9)	630.9 (±55)	3202.3 (±176)

## Conclusion

We developed the novel integrated thermophilic fermenter and dual anion exchange membrane bioelectrochemical system in order to separate volatile fatty acids and preserve ammonia, alkalinity, pH and buffering capacity during real thermophilic fermentation of food waste. Biohydrogen production increased by 632 mL L^−1^ rather than control. Furthermore, 50 mL cathodic hydrogen was produced simultaneously. Volatile fatty acids production increased in the integrated system rather than control. Ammonium concentration increased because of preventing the passage of ammonium through AEM, which was useful for enhancing biohydrogen production at lower ammonium concentrations due to the avoidance of nutrient restriction.

## Additional information

**Introduction.** Many researchers are interested in utilizing a renewable and sustainable energy made by bacteria for separating unwanted materials [15], salts [16], and volatile fatty acids [7] from water and wastewater. However, these studies have not tested the separation of volatile fatty acids, which inhibits bacterial activities, from thermophilic fermenter. Our hypothesis is a integrated thermophilic fermenter and dual anion exchange membrane bioelectrochemical system can be used to separate volatile fatty acids during thermophilic fermentation and at the same time to enhance biological and cathodic hydrogen production.

## Declaration of Competing Interest

The authors declare that they have no known competing financial interests or personal relationships that could have appeared to influence the work reported in this paper.

## Appendix Supplementary materials

Image, application 1
