# Supplementary material for: Increased biological and cathodic hydrogen production using a novel integrated thermophilic fermenter and dual anion exchange membrane bioelectrochemical system
Source: MethodsX. 2022 Jun 23;9:101770. doi: 10.1016/j.mex.2022.101770 (PMC9270241; doi:10.1016/j.mex.2022.101770)
Supplement: Supplementary file 1 [file mmc1.docx]

**Supplementary**

| **Title** | Increased biological and cathodic hydrogen production using a novel integrated thermophilic fermenter and dual anion exchange membrane bioelectrochemical system |
| --- | --- |
| **Authors** | Nadali Alavi ^a, b^, Monireh Majlessi^a, b^, Nazak Amanidaz ^a, b^*. Mirzaman Zamanzadeh^c^ Mohammad Rafiee^a, b^ |
| **Affiliations** | ^a^ Environmental and Occupational Hazards Control Research Center, Shahid Beheshti University of Medical Sciences, Tehran, Iran.  ^b^ Department of Environmental Health Engineering, School of Public Health and safety, Shahid Beheshti University of Medical Sciences, Tehran, Iran.  ^c^ Department of Environmental Health Engineering, School of Public Health, Tehran University of Medical Sciences, Tehran, Iran. |
| **Corresponding Author’s email address** | [amanidaz_n@yahoo.com](mailto:amanidaz_n@yahoo.com) |
| **Keywords** | - Biohydrogen, Cathodic H_2_, Inhibitors recovery, Bipolar electrodialysis, Bio electrochemical, Double anion exchange membrane |
| **Co-Submission** | *Co-Submission*  <https://doi.org/10.1016/j.jclepro.2021.129887> |

Total number of Figures: **7**

| B | A |
| --- | --- |
| 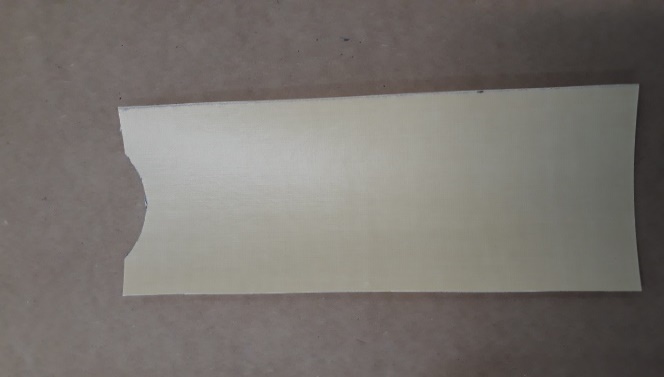 | 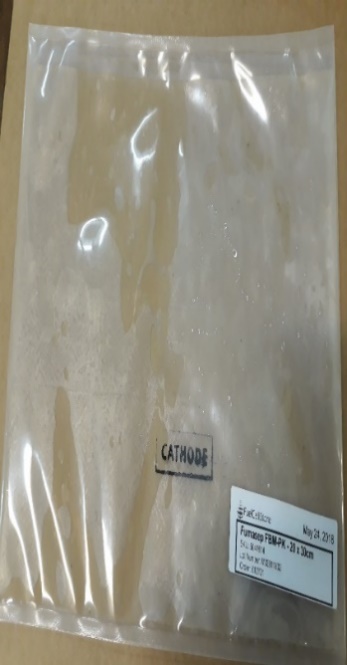 |
| D | C |
| 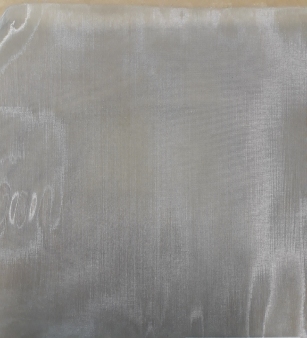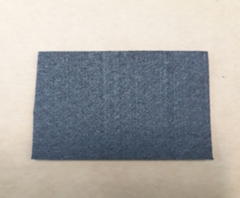 | 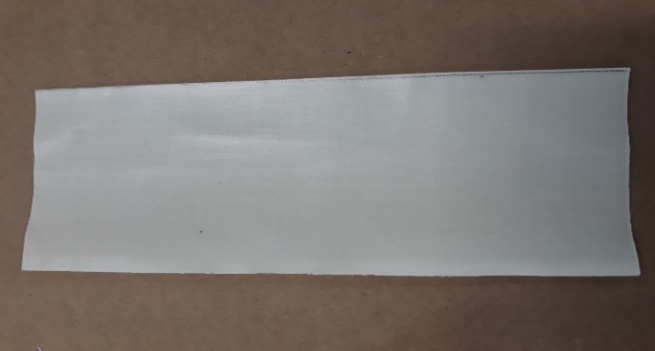 |

**Fig S1**. (A) Bipolar membrane (BPM: fumasep FBM, FuMA-Tech GmbH, Germany). (B) Cation exchange membrane (CMI 7000, Membrane international, NJ). (C) Anion exchange membrane (AMI 7001, Membrane international, NJ). (D). Carbon felt and stainless-steel mesh (Wire diameter 0.24 mm and aperture 1.57 mm, The Mesh Company, United Kingdom).

| B | A |
| --- | --- |
| 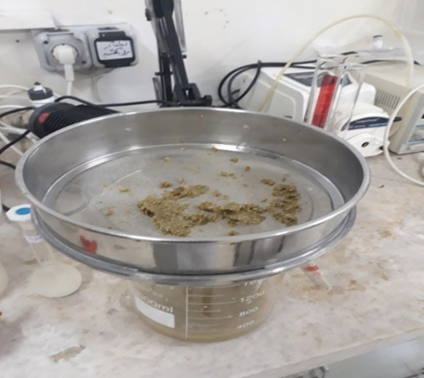 | 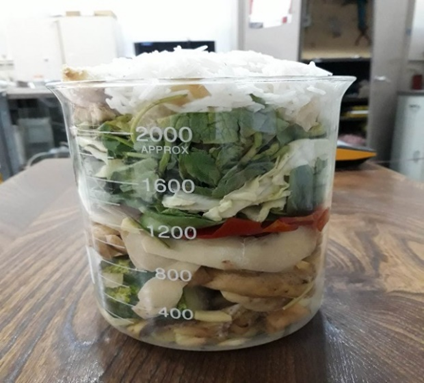 |

**Fig S2**. (A) Primary food waste with a certain percentage of each type of waste (B) Food waste passing through the 0.5 mm sieve..


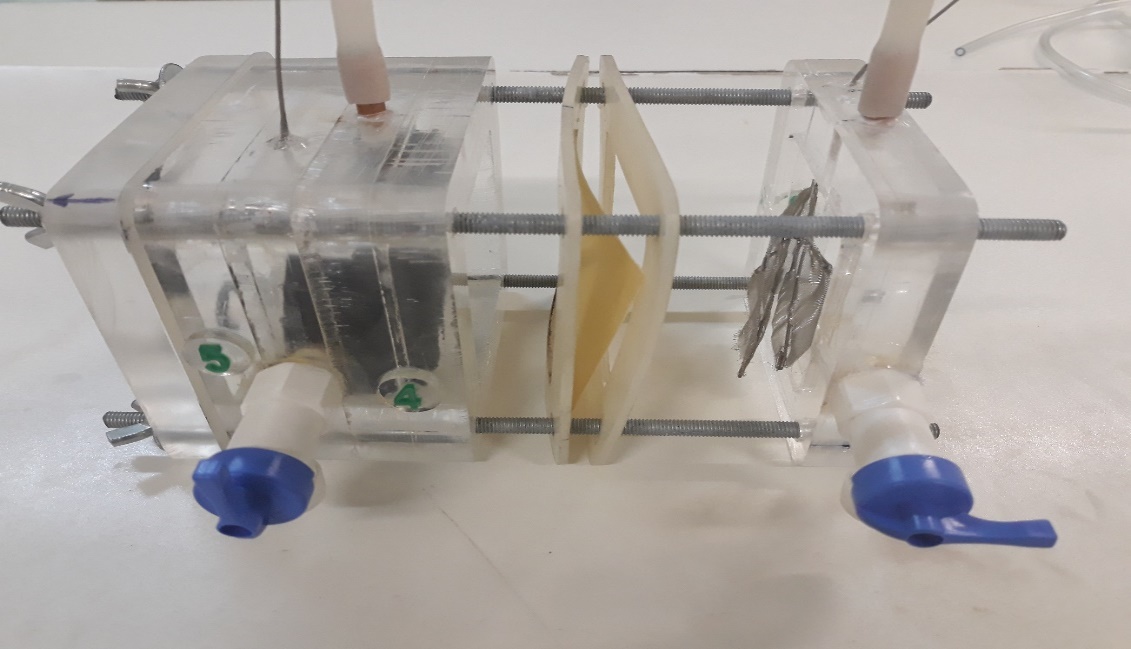


**Fig S3**. The microbial fuel cell system setup.


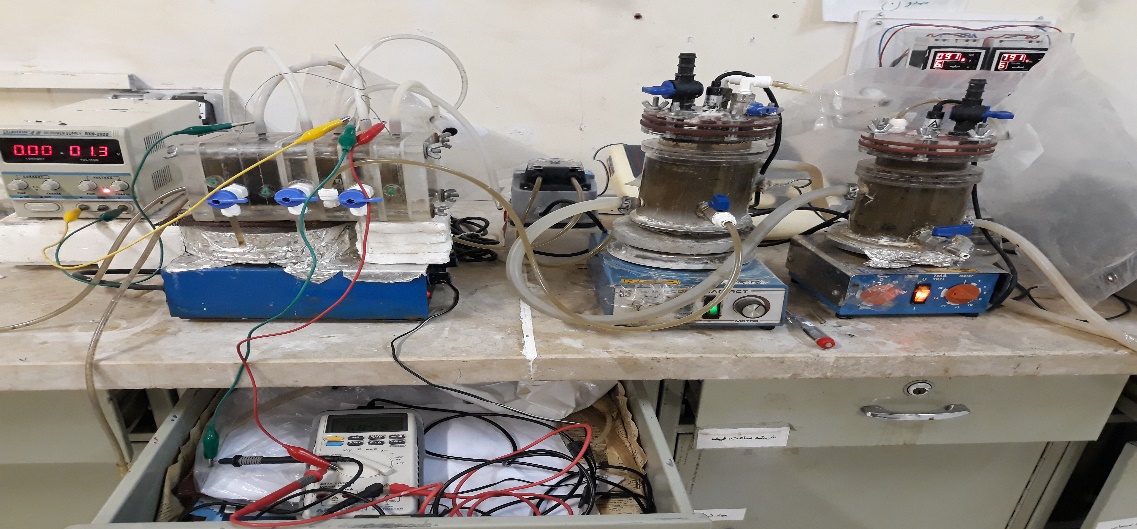


**Fig S4**. The thermophilic fermenter – dual anion exchange membrane bio electrochemical system


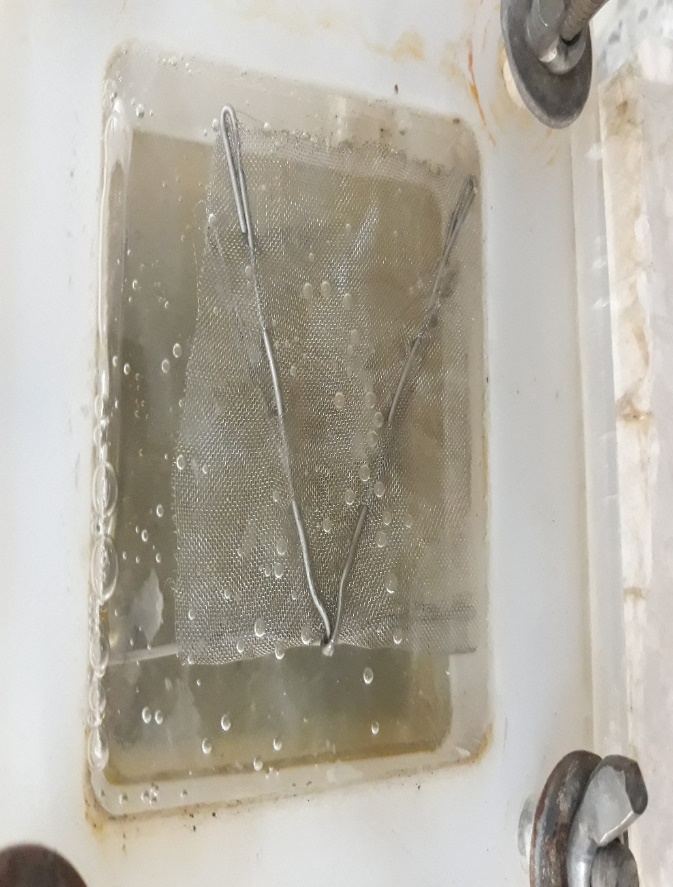


**Fig S5**. The cathodic hydrogen production in the cathode chamber.


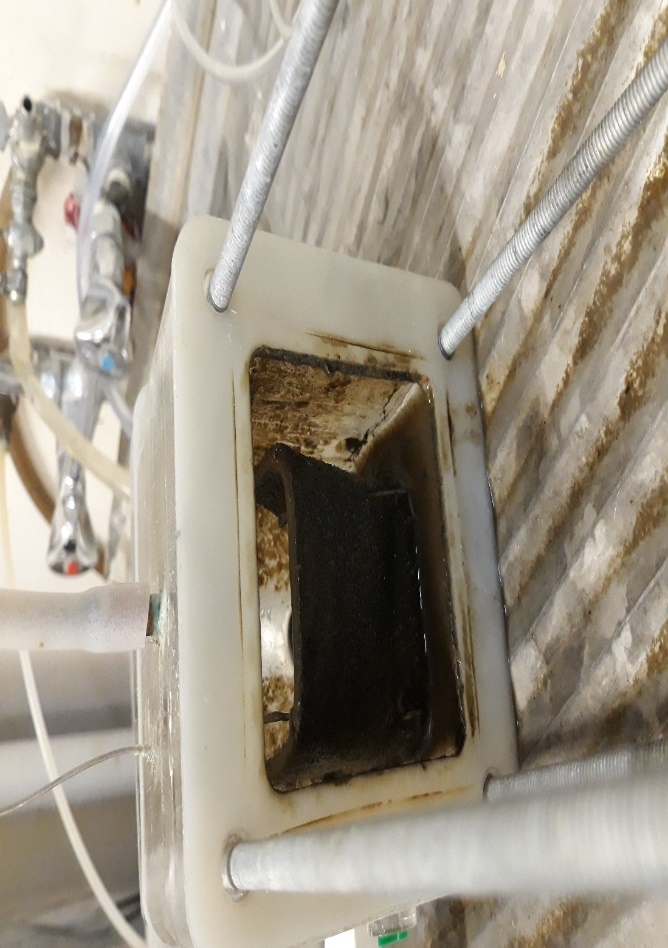


**Fig S6**. The growsed biofilme on the carbon felt in the anode chamber


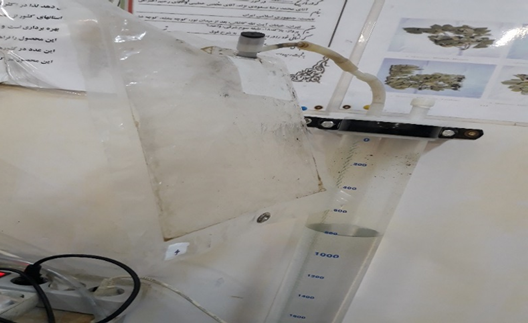


**Fig S7**. Water displacement to measure gas volume
